# Pentoxifylline changes the balance of immune cell population in breast tumor-infiltrating lymphocytes

**DOI:** 10.1007/s12032-023-02034-5

**Published:** 2023-05-06

**Authors:** Mohammad Hossein Kazemi, Mahdieh Shokrollahi Barough, Zahra Momeni-Varposhti, Alireza Ghanavatinejad, Ali Zarehzadeh Mehrabadi, Behnam Sadeghi, Reza Falak

**Affiliations:** 1 Department of Immunology, School of Medicine, Iran University of Medical Sciences, Tehran, Iran; 2 ATMP Department, Breast Cancer Research Center, Motamed Cancer Institute, ACECR, P.O. BOX: 15179/64311, Tehran, Iran; 3 Hematopoietic Stem Cell Research Center, Shahid Beheshti University of Medical Sciences, Tehran, Iran; 4 Department of Immunology, Pasteur Institute of Iran, Tehran, Iran; 5 Immunology Research Center, Institute of Immunology and Infectious Disease, Iran University of Medical Sciences, Tehran, Iran; 6 Translational Cell Therapy Research (TCR), Division of Pediatrics, Department of Clinical Science, Intervention and Technology (CLINTEC), Karolinska Institutet, Stockholm, Sweden

**Keywords:** Breast cancer, Tumor-infiltrating lymphocyte, Pentoxifylline, Regulatory T cells, Cytotoxic TILs

## Abstract

Immunotherapy utilizing tumor-infiltrating lymphocytes (TILs) is a promising approach for cancer treatment. Pentoxifylline (PTXF), a xanthine derivative, exhibits antitumor properties. This study aimed to investigate the impact of PTXF on the phenotype and function of TILs and splenocytes in a triple-negative breast cancer (TNBC) mouse model. TNBC was subcutaneously induced in BALB/c mice, followed by nine intraperitoneal injections of 100 mg/kg PTXF. TILs were then isolated by enzymatic digestion of tumors and cocultured with 4T1 cells. The proportion of regulatory *T* cells (Tregs) and cytotoxic T cells in TILs and splenocytes was assessed using flow cytometry. Transforming growth factor (TGF)-*β* and interferon (IFN)-*γ* production in TILs and splenocytes cultures was measured by ELISA. Relative expression of *t-bet*, *foxp3*, *gata-3*, and *ror-γt* in TILs and splenocytes was evaluated using real-time PCR. Tumor growth in PTXF-treated mice was significantly lower than that in the controls (*P* < 0.01). The frequency of regulatory and cytotoxic TILs in PTXF-treated mice was approximately half (*P* < 0.01) and twice (*P* < 0.05) that of the control group, respectively. The level of TGF-*β* and IFN-*γ* in the supernatant of PTXF-treated TILs was decreased and increased, respectively (*P* < 0.05). The relative expression of *t-bet* and *foxp3* in the PTXF-treated mice compared to controls was increased and decreased, respectively (*P* < 0.05). Changes in the immune cell balance were less significant in the spleen compared to the TILs. PTXF treatment could limit the tumor growth and modify the regulatory-to-cytotoxic TILs ratio, as well as cytokine balance of TILs, in favor of antitumor responses.

## Introduction

Breast cancer (BC) is the most commonly diagnosed cancer among women (25% of all women’s cancers) and the leading cause (15%) of cancer-related mortality in women [1]. Triple-negative BC (TNBC) is the most aggressive and deadliest type of BC, responsible for 15% of all BC cases, predominantly affecting young women [2]. This type of BC does not express hormone receptors and, due to its heterogeneity, has a poor response to conventional therapies [3]. The high mutation rate of TNBC makes it the most immunogenic type of BC, characterized by a relatively high degree of lymphocyte infiltration, ranging from 20 to 60% [4, 5]. In this context, immunotherapy using tumor-infiltrating lymphocytes (TILs) represents a promising approach for treating TNBC patients [6]. TILs are a crucial indicator of tumor immunogenicity and represent a prognostic factor in many cancers [7]. In BCs, the positive correlation between CD8^+^ TILs and improved clinical outcomes of cancer treatment has been extensively established [6]. Contrarily, when the majority of TIL populations are immunosuppressive cells such as regulatory *T* cells (Tregs) or exhausted immune cells, TILs might be associated with an unfavorable prognosis [8]. TIL therapy is a type of personalized immunotherapy approach that, in addition to its targeted antitumor effects, has minimal off-target toxicities [7]. Recent clinical trials have demonstrated favorable outcomes in patients with chemotherapy-resistant melanoma following immunotherapy using interleukin (IL)-2-stimulated TILs [9]. However, TIL therapy in BC is not well-studied and requires extensive research efforts. The high proportion of immunosuppressive cells in BC-derived TILs might be one of the significant hurdles to TIL therapy in this setting [6]. Therapeutic interventions aimed at decreasing the number of immunosuppressive cells in TILs may potentially enhance the effectiveness of TIL therapy. Tregs play a crucial role in immune regulation by direct binding to immune cells and secreting inhibitory cytokines such as transforming growth factor (TGF)-*β* [10].

Pentoxifylline (PTXF) is a xanthine derivative with three methyl groups that has demonstrated antitumor properties [11]. The US Food and Drug Administration (FDA) has approved the use of PTXF for peripheral arterial diseases due to its ability to improve red blood cell deformities, enhance oxygen delivery, and dilate peripheral arteries [12]. PTXF, like other methylated xanthines, exerts inhibitory effects on phosphodiesterases, resulting in increased intracellular levels of cyclic adenosine monophosphate (cAMP) [11]. According to a recent study, PTXF is able to improve antitumor responses without much concern about autoimmunity [13]. Despite its potential, few studies have assessed the impact of PTXF on TILs, and the effects of this drug on immune cells remain controversial [13–16]. In this study, we sought to determine the effects of PTXF on the phenotype and function of TILs and splenocytes in a mouse model of TNBC.

## Materials and methods

### Tumor induction and pentoxifylline treatment

The study obtained ethical approval from the Iran University of Medical Sciences, Tehran, Iran (IR.IUMS.FMD.REC.1397.220). The mouse model of TNBC was induced by subcutaneous injection of 10^5^ 4T1 cells (ATCC code: CRL-2539) into the flanks of 12 female BALB/c mice between 6 and 8 weeks of age. The tumor was induced in the flank to ensure adequate size for obtaining enough TILs. Orthotropic tumors in the mammary fat pad cannot gain enough size because it causes problems in mouse movement. Although tumors were allowed to grow to reach enough TILs, mice were humanely euthanized if their tumor size reached 1800 mm^3^ or if any signs of suffering were observed in mice. The mice were housed in a standard animal research facility center. Once the tumors became palpable, six mice received nine intraperitoneal injections of 100 mg/kg PTXF (dissolved in PBS) on days 10,11,12,13,15,17,19, 21, and 23. Six mice in the control groups received PBS as the vehicle. Tumor dimensions were measured blindly by two independent investigators every three days using a digital caliper and tumor sizes were calculated using the formula *π*/6 × length × width × height [17, 18]. It is worth noting that the dose and timing of treatment were determined based on the literature and pilot studies [13, 19–24]. The selected dose was evaluated for any adverse effects on healthy mice before the main study. The mice were observed daily for any abnormal symptoms, unwanted bleeding, and weight loss.

### TIL and splenocyte isolation

Mice were euthanized on the day after the last injection. Tumor digestion and TIL isolation were performed as previously described [14]. Briefly, the dissected tumors were floated in RPMI-1640 (Gibco, US) with 1X PenStrep solution (100 IU/mL Penicillin and 100 µg/mL Streptomycin) (Gibco, US) and mechanically cut into 1–2 mm^2^ pieces using scalpel blades. Then, the tumor pieces were incubated for 40 min in RPMI-1640 containing 0.2% collagenase IV (Gibco, US) and 10 U/mL DNase I (Sigma-Aldrich, US) in a shaker incubator at 37 °C and 90 rpm. Single cells were obtained by passing the digested solution through a 70 µm cell strainer (SPL, South Korea). Mononuclear cells were harvested by centrifuging the suspension on the Ficoll 1.077 (GE Healthcare, Sweden) gradient at 800×g for 20 min. Spleens were ruptured in the RPMI-1640 medium, and the solution was passed through a 70 µm cell strainer. Splenocytes were isolated using Ficoll density gradient centrifugation, as described earlier.

### Flow cytometry

TILs and splenocytes were superficially stained with FITC-conjugated anti-CD3 (clone: 145-2C11, BD Bioscience, US), anti-CD25 (clone: 7D4, BD Bioscience, US), PE-Vio770-conjugated anti-CD4 (clone: REA604, Miltenyi Biotec, Germany), and PE-conjugated anti-CD8 (clone: 53–6.7, BD Bioscience, US) antibodies. The concentration of each conjugated antibody was one microgram per 10^5^ cells in PBS containing 2% fetal bovine serum (FBS). Following incubation at 4 °C for 30 min, the excessive antibodies were removed by washing the cell suspensions. For intracellular staining of FOXP3, cells were fixed and permeabilized using True Nuclear Transcription Factor Buffer Set (BioLegend, San Diego, CA), and PE-conjugated anti-FOXP3 antibody (clone: MF23, BD Biosciences, CA) was added to cells according to the manufacturer protocol. All analyses were carried out on live cells using Zombie NIR Fixable Viability Kit (BioLegend, San Diego, CA). Gating strategies were based on unstained and fluorescent minus one (FMO) controls. Flow cytometry analysis was conducted using an Attune NXT flow cytometer (Invitrogen, US), and data were analyzed by FlowJo software (Tree Star).

### Degranulation assay

TILs or splenocytes were cocultured in triplicates at a concentration of 5 × 10^5^ cells/mL with 4T1 cells at the ratio of 5:1 in 48-well culture plates (SPL, South Korea) containing the RPMI-1640 with 1X PenStrep solution, 10% FBS (Gibco, US) and 150 U/mL IL-2 (Miltenyi Biotec, Germany), known as complete medium. The optimum coculture ratio was obtained based on pilot studies. TILs or splenocytes were also cultured alone as a negative (unstimulated) control. For positive (stimulated) control, TILs or splenocytes were activated by adding 1 µL/well of Cell Activation Cocktail (Biolegend, San Diego, CA), containing 0.5 μM Phorbol Myristate Acetate and 7 μM Ionomycin (PMA-I) without Brefeldin A. One microgram of APC-conjugated CD107a (clone: 1D4B, Biolegend, Sandiego, CA) was added to each well from the beginning of the coculture. Following one hour of incubation in a cell-culture incubator (at 37 °C and 5% CO2), 1 µL/mL of Monensin Solution 1000x (Biolegend, San Diego, CA) was added to wells and incubated for an extra 5 h. As described earlier, cells were harvested, washed, and surface stained for CD3 and CD8.

### Cytokine assay

TILs or splenocytes were cocultured in triplicates at a concentration of 5 × 10^5^ cells/mL with 4T1 cells at the ratio of 5:1 in 96-well culture plates for 12 h, as described in the previous section. TILs and splenocytes were cultured alone as negative (unstimulated) control. For positive (stimulated) control, TILs or splenocytes were stimulated by Cell Activation Cocktail (PMA-I) for 6 h. After the incubation period, supernatant samples were carefully collected for cytokine assay. The levels of interferon (IFN)-*γ* and TGF-*β* were quantified using the enzyme-linked immunosorbent assay (ELISA) kits (eBioscience, San Diego, CA), according to the manufacturer's recommended protocol.

### Gene expression

Total RNA was extracted from TILs and splenocytes using the SinaPure RNA extraction kit (SinaClon BioScience, IRAN), according to the manufacturer. The concentration and purity of extracted RNA were evaluated by a nanodrop spectrophotometer (2000c, ThermoFisher Scientific, US) and gel electrophoresis. One microgram of extracted RNA was reverse-transcribed to cDNA using RevertAid First Strand cDNA Synthesis Kit (ThermoFisher Scientific, US) in a Peqstar 96X Thermal Cycler (Peqlab, Germany), based on manufacturer protocol. The relative expression of *t-bet*, *gata-3*, *ror-γt*, and *foxp3* genes were evaluated using the SYBR Green real-time PCR method. The employed primer sequences are presented in Table 1.

**Table 1:** Employed primer sequences

Target gene	Sequence
*t-bet*	Forward: 5′- TCAACCAGCACCAGACAGAG-3′
	Reverse: 5′- CCACATCCACAAACATCCTG-3′
*gata-3*	Forward: 5′- AAGGCAGGGAGTGTGTGAAC-3′
	Reverse: 5′- TCGCTTGGGCTTGATAAGGG-3′
*ror-γt*	Forward: 5′- TGCAAGACTCATCGACAAGG-3′
	Reverse: 5′- AGGGGATTCAACATCAGTGC-3′
*foxp3*	Forward: 5′- ATCTCCTGGATGAGAAAGGCAAGG-3′
	Reverse: 5′- TGTTGTGGAAGAACTCTGGGAAGG-3′
*gapdh*	Forward: 5′- GAGTCAACGGATTTGGTCGT-3′
	Reverse: 5′- GACAAGCTTCCCGTTCTCAG-3′

Real-time PCR was performed with Rotor-Gene Q (Qiagen, Korea). The total volume in each tube was 20 μL, containing 1 μL cDNA, 10 μL of SYBR Green Master Mix (Ampliqon, Denmark), and 2 μL of both reverse and forward primers. The thermal cycling protocol comprised 1 cycle of 95 °C for 5 min, followed by 40 cycles of 95 °C for 15 s, and 56–60 °C for 40 s. Glyceraldehyde 3-phosphate dehydrogenase (gapdh) was considered as the reference gene, and the 2^−(ΔΔCT)^ method was employed to evaluate the relative expression level.

### Statistical analyses

The experiments were conducted in triplicate. Additionally, the reproducibility of results was confirmed via a separate in vivo experiment and several pilot studies. Based on the normal distribution of data, one-way ANOVA (Tukey’s multiple comparisons) and Student *T*-test were employed to compare means. The results were analyzed and illustrated using GraphPad PRISM 8 with *P*-value < 0.05 significant index.

## Results

### Pentoxifylline delays TNBC growth

**Fig. 1:**
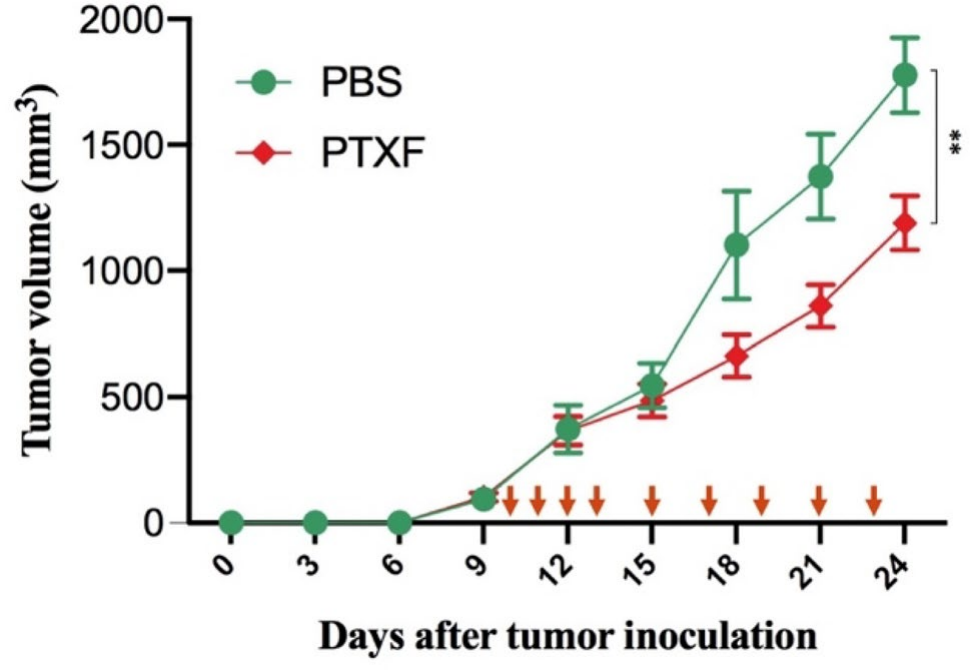
Tumor volume in the control and treated groups. Tumor-bearing mice were divided into two groups (6 mice in each group) and received 9 intraperitoneal injections of either 100 mg/kg PTXF (red line) or PBS (green line). Red arrows show the injections. Their tumor size was measured every three days using a digital caliper and demonstrated as mean ± SD. The Mann–Whitney *U* test was employed to compare the results between the two groups.

Experimental animals were divided into two groups consisting of PTXF-treated and control (receiving PBS). The mean tumor size was approximately the same in both groups until day 12. Subsequently, the mean tumor size in the PTXF-treated group exhibited significantly less growth than the control group, with a statistically significant difference observed on day 24 (*P* < 0.01) (Fig. 1).

### Pentoxifylline decreases Treg proportion in TILs but not in splenocytes

**Fig. 2:**
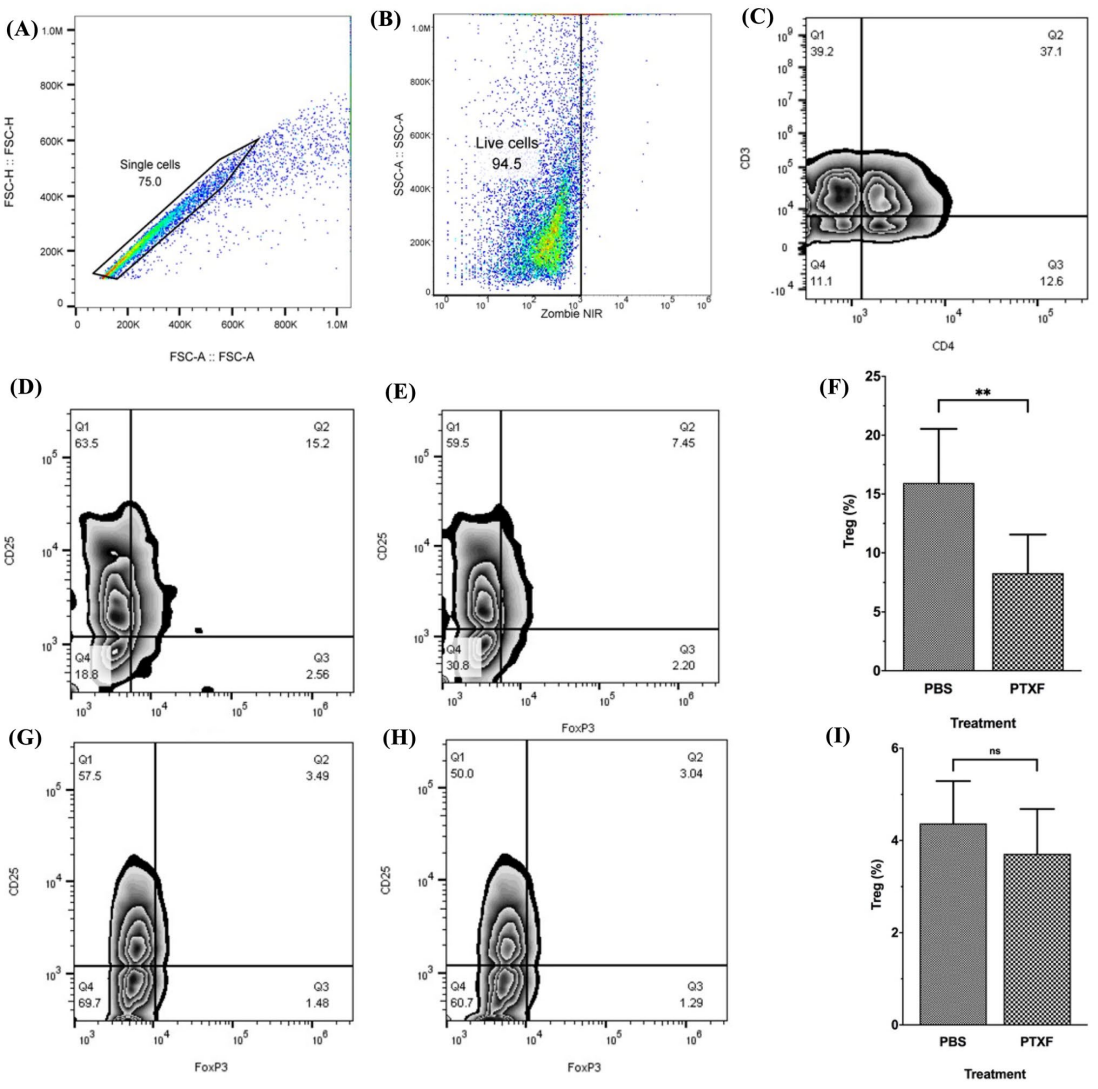
Effects of pentoxifylline on the Treg proportion in TILs and splenocytes. TILs and splenocytes were isolated from all PTXF-treated (6 mice) and control mice (6 mice). Flow cytometry analyses were performed on single (**A**) live (**B**) cells. Due to the Ficoll separation, most of the cells were viable. **C** shows the gating strategy to select CD4^+^ *T* cells. Among CD4^+^ *T* cells, CD25^+^ and FOXP3^+^ cells were gated as Treg cells. **D** and **E** representatively show the gating on CD25^+^ and FOXP3^+^ cells in the TILs of PTXF-treated (2D) and control (2E) mice. Also, **G** and **H** representatively show the gating on CD25^+^ and FOXP3^+^ cells in the splenocytes of PTXF-treated (2G) and control (2H) mice. **F** and **I** show statistical comparisons between the mean percentage of Treg cells in TILs (2F) and splenocytes (2I) of the two mice groups. The comparisons between two groups were performed using Student *T*-test. *TIL* Tumor-infiltrating lymphocytes; *Tregs* Regulatory *T* cells; *PTXF* Pentoxifylline; *PBS* Phosphate buffer saline; *n.s.* Not significant; ** = *P* < 0.01

Following the isolation of TIL and splenocytes, the proportion of Tregs was examined by flow cytometry. Analyzes were performed on single live cells (Fig. 2A and B). Tregs were identified by gating CD4^+^ CD25^+^ FOXP3^+^ cells, as shown in Fig. 2.

The Treg proportion in TILs and splenocytes of PTXF-treated and control mice were compared together (Fig. 2F and I). Results showed that the ratio of Tregs in TILs of PTXF-treated mice was significantly lower than that of the control group (*P* < 0.01) (Fig. 2D–F). Interestingly, the mean percentage of Treg TILs in the PTXF-treated mice was nearly half of the Treg percentage in the TILs of controls. Although the proportion of Tregs in splenocytes of PTXF-treated mice was lower than that of the control group, the difference between these two groups was not statistically significant (*P* = 0.3) (Fig. 2G–I).

### Pentoxifylline increases cytotoxic TILs

**Fig. 3:**
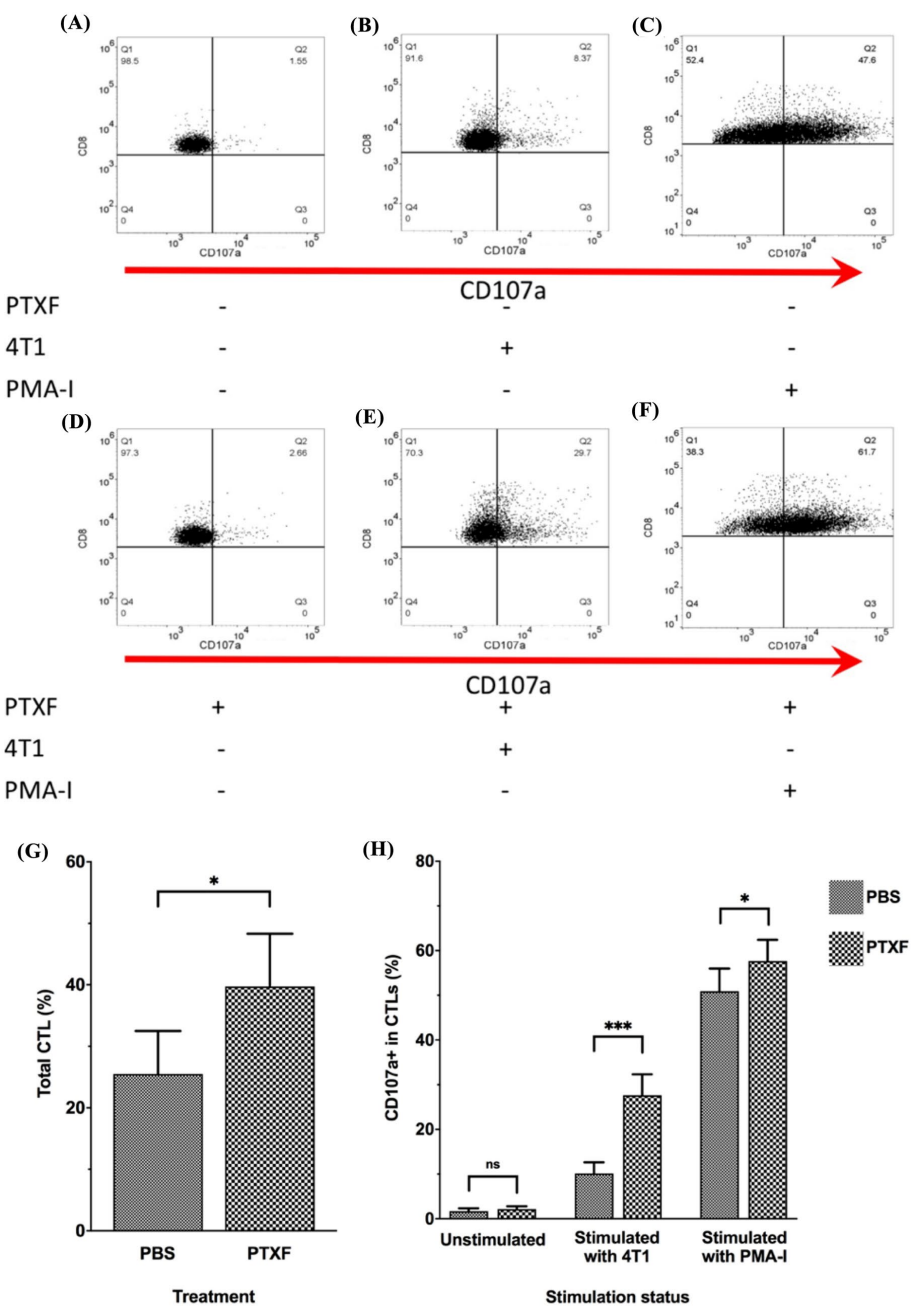
Effects of pentoxifylline on the total and cytotoxic CD8^+^ *T* cells in TILs. TILs were isolated from all PTXF-treated (6 mice) and control mice (6 mice). The isolated TILs were divided into three groups. The first group of TILs was cultured alone (unstimulated control). The second TIL group was cocultured with 4T1 cells (TNBC cell line), and the third TIL group was stimulated with PMA-I (stimulated control). TILs were then examined by flow cytometry. First, CD3^+^ and CD8^+^ cells were selected as total CD8^+^ *T* cells. Then, the CD107a^+^ cells were considered degranulated (cytotoxic) CTLs (**A**–**F**). **G** shows the bar chart comparing total CD8^+^ TILs between PTXF-treated and control groups. **H** shows the bar chart comparing CD107a^+^ cells in CD8^+^ TILs between PTXF-treated and control groups. The comparisons between two groups were performed using Student *T*-test. *TIL* Tumor-infiltrating lymphocytes; *CTLs* Cytotoxic *T* lymphocytes; *PTXF* Pentoxifylline; *PBS* Phosphate buffer saline; *PMA-I* Phorbol Myristate Acetate (PMA) and Ionomycin; *n.s.* Not significant; * = *P* < 0.05; *** = *P* < 0.001

In the next step, the proportion of total CD8^+^ *T* cells in TILs of PTXF-treated mice and control mice was compared (Fig. 3). To identify the cytotoxic *T* lymphocytes (CTLs), we performed a degranulation assay. TILs were cultured alone (unstimulated control), cocultured with 4T1 cells (TNBC cell line), and cultured in the presence of PMA-I (stimulated control) (Fig. 3A–F). The CD107a^+^ CD8^+^ *T* cells were considered effector (degranulated) CTLs and compared between the two groups.

Our results showed that the mean percentage of total CD8^+^ *T* cells in TILs derived from the PTXF-treated group was about 40%, which was significantly higher than that of the control group (*P* < 0.05) (Fig. 3.G). The unstimulated TILs showed the lowest CD107a^+^ cells, about 1–2% of CTLs, with no significant difference between the two groups (Fig. 3H). In the part where the TILs were cocultured with the 4T1 cells, it was observed that the ratio of CD107a^+^ CTLs in the PTXF-treated group was significantly (about 2.5 times) higher than that in the control group (*P* < 0.001) (Fig. 3H). The highest percentage of CD107a^+^ CTLs was observed in the part where TILs were stimulated with PMA-I. In this case, the ratio of CD107a^+^ CTLs in the PTXF-treated group was significantly higher than that of the control group (*P* < 0.05) (Fig. 3H).

### Pentoxifylline does not increase cytotoxic *T* cells in splenocytes

**Fig. 4:**
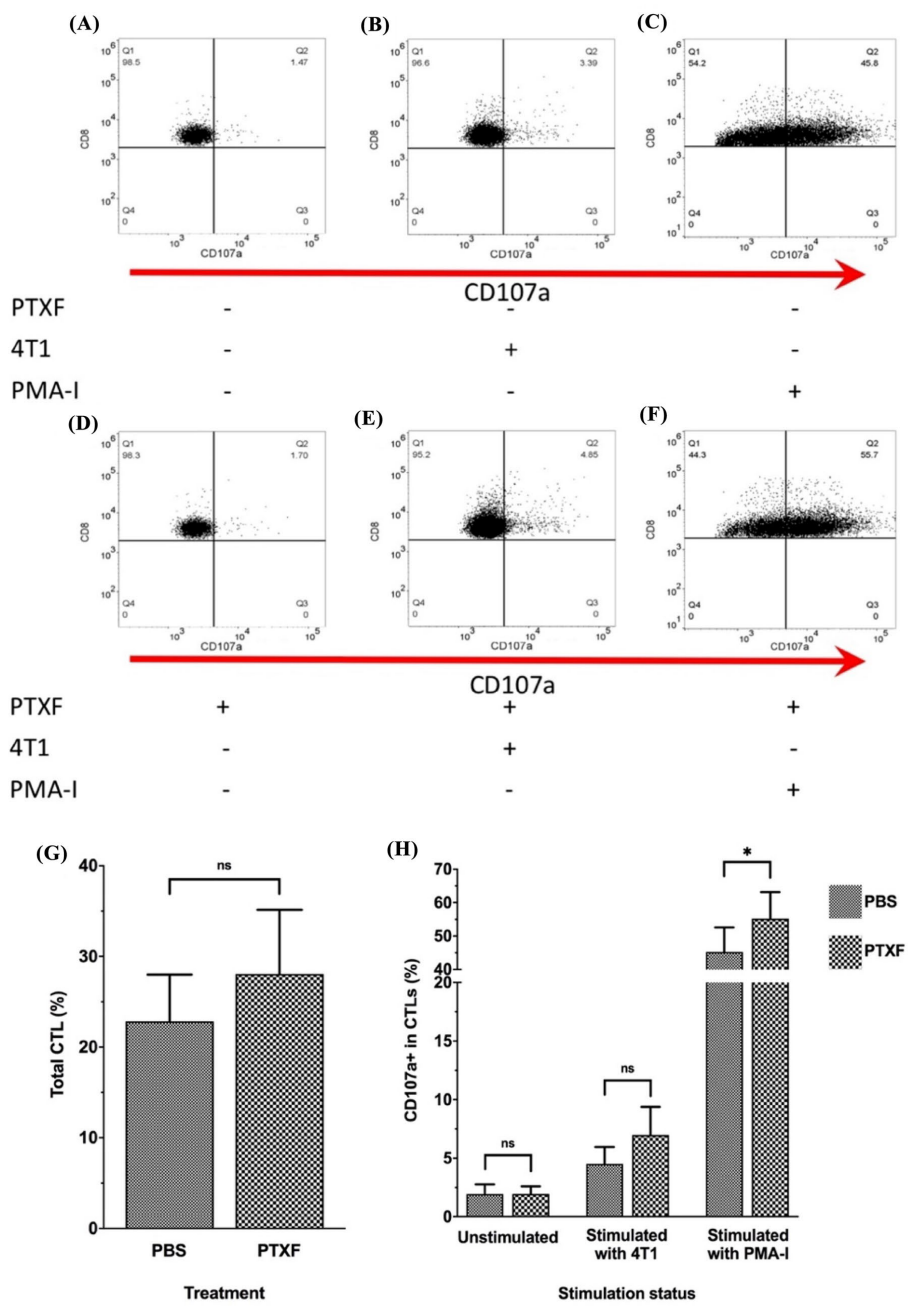
Effects of pentoxifylline on the total and cytotoxic CD8^+^ *T* cells in splenocytes. Splenocytes were isolated from all PTXF-treated (6 mice) and control mice (6 mice). The isolated splenocytes were divided into three groups. The first group was cultured alone (unstimulated control). The second splenocyte group was cocultured with 4T1 cells (TNBC cell line), and the third group was cultured in the presence of PMA-I (stimulated control). Splenocytes were then examined by flow cytometry. First, CD3^+^ and CD8^+^ cells were selected as CD8^+^ *T* cells. Then, the CD107a^+^ cells were considered degranulated (cytotoxic) CTLs (**A**–**F**). **G** shows the bar chart comparing total CD8^+^ splenocytes between PTXF-treated and control groups. **H** shows the bar chart comparing CD107^+^ cells in CD8^+^ splenocytes between PTXF-treated and control groups. The comparisons between two groups were performed using Student *T*-test. *CTLs* Cytotoxic *T* lymphocytes; *PTXF* Pentoxifylline; *PBS* Phosphate buffer saline; *PMA-I*. Phorbol Myristate Acetate (PMA) and Ionomycin; *n.s.* Not significant; * = * P*< 0.05

In addition to TILs, we evaluated and compared the proportion of total CD8^+^ *T* cells and CD107a^+^ CD8^+^ *T* cells in splenocytes of PTXF-treated and control mice (Fig. 4). The gating strategy and coculture system were consistent with those employed for TILs (Fig. 4A–F).

The mean percentage of total CD8^+^ *T* cells in splenocytes derived from the PTXF-treated group was marginally and insignificantly higher than that of the control group (*P* = 0.17) (Fig. 4G). The unstimulated splenocytes showed the lowest proportion of CD107a^+^ cells, comprising 2% of CD8^+^ *T* cells, with no statistically significant difference between the two groups (Fig. 4H). In the coculture system with 4T1 cells, the ratio of CD107a^+^ CTLs in the PTXF-treated group was slightly greater than that in the control group. However, the difference between the two groups was near the significance level (*P* = 0.07) (Fig. 4H). The highest percentage of CD107a^+^ CTLs was observed in the PMA-I-stimulated splenocytes, wherein the ratio of CD107a^+^ CTLs in the PTXF-treated group was significantly higher than that of the control group (*P* < 0.05) (Fig. 4H).

### Pentoxifylline alters the cytokine balance of TILs in favor of antitumor responses

To investigate the impact of PTXF on cytokine secretion from TILs and splenocytes, we cultured these cells alone (unstimulated control), cocultured them with 4T1 cells (TNBC cell line), and stimulated them with PMA-I (stimulated control). The culture supernatant was subjected to cytokine assay. The amounts of IFN-*γ* as an antitumor cytokine and TGF-*β* as a tumor-promoting cytokine were quantified by ELISA.

**Fig. 5:**
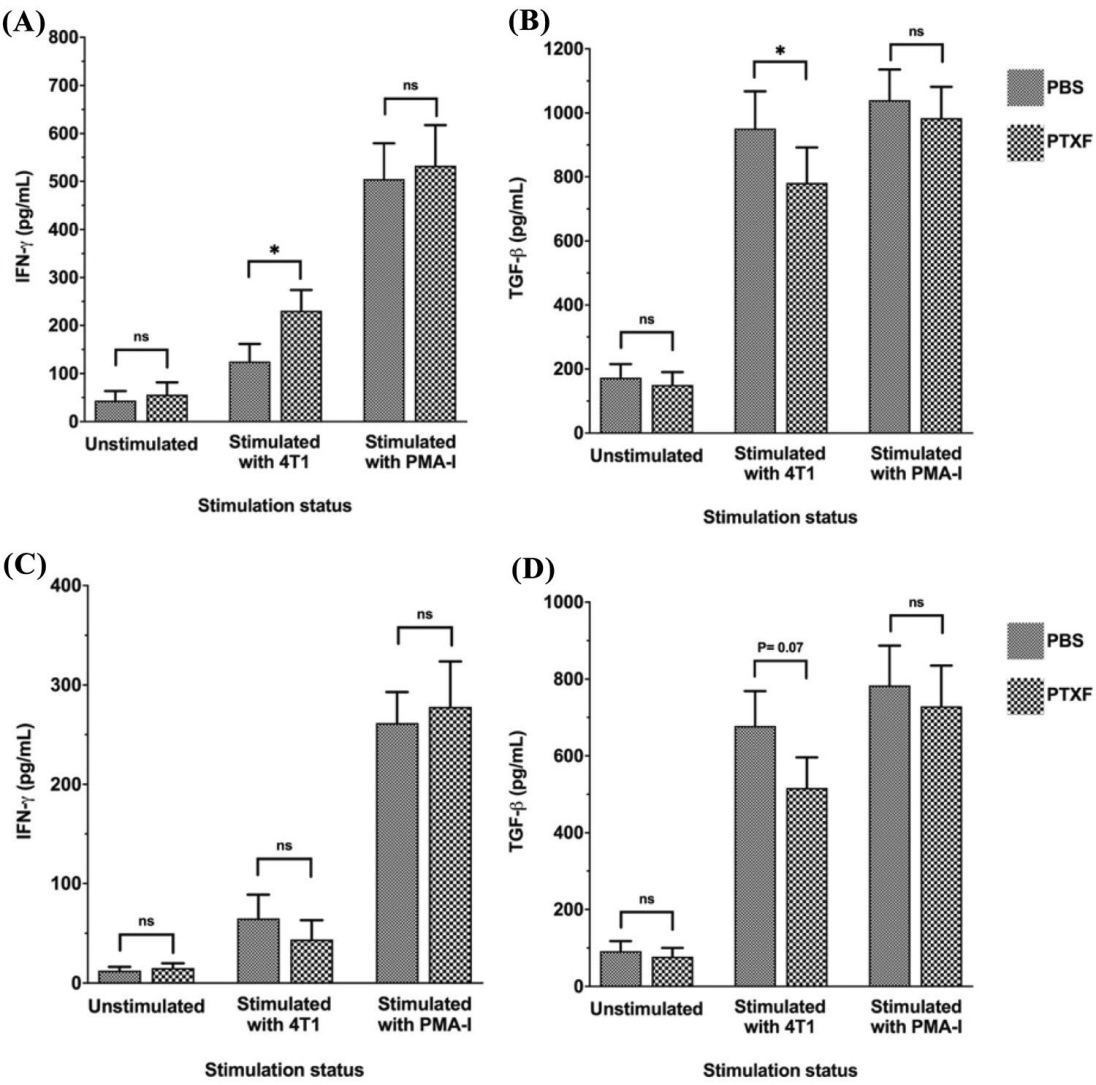
Effects of pentoxifylline on the cytokine balance of TILs and splenocytes. TILs and splenocytes were isolated from all PTXF-treated (6 mice) and control mice (6 mice). The isolated TILs or splenocytes were divided into three groups. The first group was cultured alone (unstimulated control). The second TIL or splenocyte group was cocultured with 4T1 cells (TNBC cell line), and the third group was cultured in the presence of PMA-I (stimulated control). Then the secretion of IFN-*γ* and TGF-*β* in the culture supernatant was assessed by ELISA. The comparisons between two groups were performed using Student *T*-test. *PTXF* Pentoxifylline; *PBS* Phosphate buffer saline; *PMA-I* Phorbol Myristate Acetate (PMA) and Ionomycin;* IFN-γ*. Interferon-gamma; *TGF-β*. Tumor growth factor-beta; *n.s.* Not significant; * = *P* < 0.05

As depicted in Fig. 5A, the levels of IFN-*γ* in the supernatant of unstimulated TILs in both PTXF-treated and control mice were low, approximately 50 pg/mL, and showed no significant difference between the two groups. However, in the supernatant of 4T1-cocultured TILs, the amount of IFN-*γ* in the PTXF-treated group was significantly higher than that in the control group (*P* < 0.05). Notably, stimulation of TILs with PMA-I led to a marked increase in IFN-*γ* in the supernatant of TILs, which was similar between the two groups (Fig. 5A).

Figure 5B shows that there was no significant difference in the TGF-*β* levels in the supernatant of unstimulated TILs isolated from PTXF-treated and control mice. However, in the supernatant of 4T1-stimulated TILs, the TGF-*β* level in the PTXF-treated group was significantly lower than that of the control group (*P* < 0.05). The level of TGF-*β* in the supernatant of PMA-I stimulated TILs in both groups showed a sharp increase compared to the unstimulated TILs, but no significant difference was observed in the TGF-β levels between the two groups of mice (Fig. 5B).

Figure 5C depicts that the level of IFN-*γ* in the supernatant of unstimulated splenocytes in both PTXF-treated and control mice was low, with no significant difference between the two groups. In the supernatant of 4T1-cocultured splenocytes, the amount of IFN-*γ* in the PTXF-treated group was slightly lower than that of the control group, but this difference was not statistically significant (*P* = 0.78). Stimulation of splenocytes with PMA-I caused a sharp increase in IFN-*γ* in the supernatant of splenocytes, with no significant difference between the two groups (Fig. 5C).

Figure 5D reveals no significant difference between the TGF-*β* levels in the supernatant of unstimulated splenocytes isolated from PTXF-treated and control mice. However, in the supernatant of 4T1-cocultured splenocytes, the TGF-*β* level in the PTXF-treated group was slightly lower than that of the control group, although this difference did not reach statistical significance (*P* = 0.07). Moreover, stimulation of splenocytes with PMA-I caused a marked increase in TGF-*β* in the supernatant of splenocytes, which was not significantly different between the two groups (Fig. 5D).

### Pentoxifylline decreases the *foxp3* and increases the *t-bet* genes expression in TILs

**Fig. 6:**
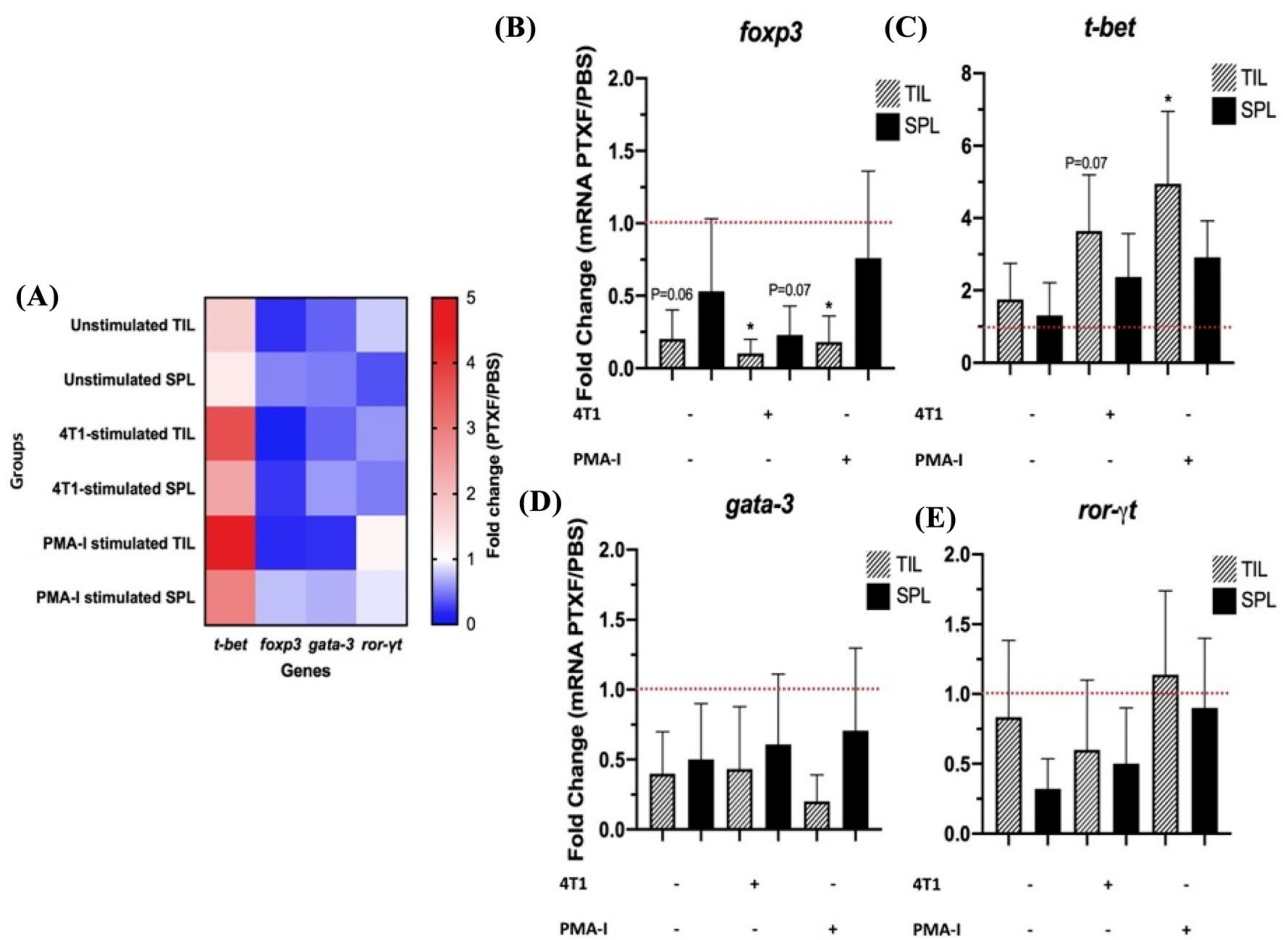
Effects of pentoxifylline on the gene expression profile of TILs and splenocytes. TILs and splenocytes were isolated from all PTXF-treated (6 mice) and control mice (6 mice). The isolated TILs or splenocytes were divided into three groups. The first group was cultured alone (unstimulated control). The second TIL or splenocyte group was cocultured with 4T1 cells (TNBC cell line), and the third group was cultured in the presence of PMA-I (stimulated control). The relative expression of *t-bet* (TH1), *foxp3* (Treg), *gata-3* (TH2), and *ror-γt* (TH17) genes was measured by real-time PCR. **A** shows the relative expression of these genes as a heatmap. The lowest relative expression is shown in blue, and the highest relative expression of genes is shown in red. **B**–**E** are bar charts demonstrating the relative gene expression changes in the PTXF-treated group compared to the control group. The comparisons between two groups were performed using Student *T*-test. *PTXF* Pentoxifylline; *PBS* Phosphate buffer saline; *PMA-I*. Phorbol Myristate Acetate (PMA) and Ionomycin; *TH* Helper *T* cell; *Treg*. Regulatory *T* cell; *n.s.* Not significant; * = *P* < 0.05

The effects of PTXF on the expression of *t-bet*, *foxp3*, *gata-3*, and *ror-γt* genes were investigated by culturing TIL and splenocytes alone, cocultured with 4T1 cells, and stimulated with PMA-I. The relative expression of these genes, which play a crucial role in the differentiation of helper *T* (TH)1, Treg, TH2, and TH17 cells, respectively, were measured, and the results are presented in Fig. 6A as a heatmap. Additionally, Fig. 6B–E shows bar charts illustrating the relative gene expression changes in the PTXF-treated group compared to the control group.

According to Fig. 6B, the relative expression of the *foxp3* gene in both TILs and splenocytes of the PTXF-treated mice decreased compared to the control group. However, this decrease was statistically significant only in 4T1-cocultured and PMA-I-stimulated TILs. Compared to the control group, the relative decrease of *foxp3* gene expression in unstimulated TILs and 4T1-cocultured splenocytes of PTXF-treated mice was close to the significance level (*P* = 0.06 and *P* = 0.07, respectively).

The relative expression of the *t-bet* gene in both TILs and splenocytes of PTXF-treated mice was increased compared to the control group, but this increase was significant only in PMA-I stimulated TILs. The difference between the two groups in terms of the *t-bet* expression in 4T1-stimulated TILs was also near the significance level (*P* = 0.07).

Figure 6D and E demonstrate that although the relative expression of *gata-3* and *ror-γt* genes in both TILs and splenocytes were lower in the PTXF-treated group than in the control group, none of the relative expression changes was statistically significant.

## Discussion

In this study, we aimed to investigate the effect of PTXF administration on TNBC-derived TILs in BALB/c mice. We first examined the effects of PTXF on tumor growth and observed that PTXF restricts the tumor growth. The PTXF effects on reducing tumor growth in mouse models of melanoma and colon cancer have been previously reported [13]. Grinberg et al. reported that PTXF could not inhibit tumor growth in immunodeficient (RAG1^−/−^) or CD8-depleted mice with melanoma [13], indicating that the inhibitory effect of PTXF on tumor growth is primarily mediated through its effect on immune cells. Fingert et al. showed in a study of human bladder and breast cancer in xenograft mice that injection of only two doses of 257 mg/kg of PTXF had the same effect as two doses of Tiotapa in restricting tumor growth [20]. Furthermore, this study suggested that the use of PTXF could dramatically enhance the effect of chemotherapy in reducing tumor growth [20].

Kamran et al. injected 9 doses of PTXF intraperitoneally into SCID-NOD mice with established human melanoma and found that PTXF dose-dependently inhibits tumor growth [23]. This group also showed that PTXF was able to reduce cancer cell migration, inhibit proliferation, and induce cell cycle arrest and apoptosis in melanoma cells [23]. These findings suggest that PTXF can limit tumor growth via several mechanisms. However, in our study, the direct effect of PTXF on the cell cycle and apoptosis of cancer cells was not investigated, which can be considered a limitation of the present study.

Various studies have reported the antitumor effects of PTXF, particularly in reducing metastasis and enhancing the effects of chemotherapy and radiation [11, 19, 21, 22, 24]. For example, Wang et al. injected 50, 100, and 200 mg/kg of PTXF intraperitoneally into different groups of xenograft mice with established human hepatocellular carcinoma for 28 days and found that tumor growth was significantly reduced up to 60% in all treatment groups compared to the control group, regardless of the PTXF dose [25]. Therefore, it may be postulated that continuous treatment with PTXF is more crucial than the dose, which requires further investigation in future studies.

In our study, we observed a significant decrease in the proportion of regulatory TILs and an increase in the percentage of CD8^+^ *T* cells in mice receiving PTXF compared to controls. However, increasing the frequency of CD8^+^ *T* cells alone may not be sufficient to determine enhanced antitumor responses. Thus, we evaluated the expression of CD107a on CD8^+^ *T* cells as a marker of their degranulation and antitumor activity. CD107a is a lysosomal protein that transiently appears on the cell surface during degranulation and is directly related to the activation and degranulation of immune cells [26, 27]. Hence, many studies have utilized CD107a evaluation as a sensitive method to investigate the activation and cell-killing capacity of CTLs [28–30].

Our results showed that the proportion of CD107a^+^ CTLs in the PTXF-treated group was about 2.5 times higher than that of the control group. The increase of CD107a^+^ CTLs in TILs that specifically cocultured with 4T1 tumor cells suggests that PTXF may have increased the proportion of tumor-reactive CTLs in the TIL population.

One of our questions was: whether the PTXF effects on immune cells were systemic or restricted to TILs? Therefore, we also studied splenocytes as a representative of systemic immune cells. Interestingly, the proportion of splenocyte-derived Tregs and CD8^+^ *T* cells in the PTXF-treated group was not significantly different from those in the control group. Furthermore, although the ratio of CD107a^+^ CTLs in the PTXF-treated group was higher than the control group, this difference was not statistically significant.

In the next step, TILs and splenocytes were cocultured specifically with 4T1 tumor cells, and the levels of IFN-*γ* as an antitumor cytokine and TGF-*β* as a tumor-promoting cytokine were analyzed. We found that the level of IFN-*γ* in the supernatant of TILs isolated from PTXF-receiving mice was significantly higher than that of the control group. Contrarily, the level of TGF-*β* in the supernatant of TILs isolated from PTXF-treated mice was significantly lower than that of the control group. These observations support the notion that PTXF treatment can improve the antitumor function of TILs. Examination of IFN-*γ* and TGF-*β* cytokines in the supernatant of splenocytes showed no significant difference between the PTXF-treated and control groups. Noteworthy, the number of seeded TILs or splenocytes for cytokine assay was same in the PTXF and control groups. Although it could be argued that the differences in cytokine levels between the PTXF and control groups might be partly attributed to differences in TIL expansion, the final amount of secreted IFN-*γ* from the same seeding number of TILs was significantly greater in the TILs of PTXF-treated mice compared to those of the control group.

Moreover, the relative expression of *t-bet*, *foxp3*, *gata-3*, and *ror-γt* genes in TILs and splenocytes showed that PTXF increased the expression of the *t-bet* gene. In contrast, the expression of the *foxp3* gene decreased after PTXF treatment. The *t-bet* is a crucial gene involved in TH1 differentiation [31]. Bleyer et al. have previously shown that PTXF upregulates *t-bet* and *ifng* genes expression while reducing the expression of *foxp3* and *tgfb1* genes [13]. These findings were consistent with our molecular and protein results, as we also observed an increase in *t-bet* gene expression and a decrease in *foxp3* gene expression following PTXF treatment. Furthermore, we found an increase in the level of IFN-*γ* and a decrease in the level of TGF-*β* cytokines following PTXF treatment that are in line with the increase in *ifng* and the decrease in *tgfb1* genes expression in the study of Bleyer and colleagues [13].

The preferential effects of PTXF on Tregs of the TME over the Tregs of splenocytes may be attributed to the distinct nature of these two cell populations. It has been shown that Tregs in the TME are activated Tregs, while Tregs in the spleen are resting Tregs [13]. Activated Tregs are more reliant on the c-Rel subtype of NF-kB, while resting Tregs are more dependent on p65 [13]. As PTXF has been previously identified as a c-Rel inhibitor [13, 32], it is possible that the preferential effect of PTXF on Tregs within the TME over systemic Tregs in the spleen may be mediated through its effect on c-Rel. Several studies have reported the inhibitory effect of PTXF on c-Rel [13, 32]. Therefore, we did not evaluate the c-Rel expression in our study. Besides, the limited difference observed in the results of splenocytes of PTXF-treated mice versus controls might be partially due to the composition of immune cells in the spleen. Despite TILs, which are predominantly composed of *T* cells, spleen has a considerable amount of B cells. The lower frequency of Tregs in the spleen compared to the TME may have influenced the results of the splenocyte analysis. Therefore, to more accurately evaluate the effect of PTXF on spleen-derived *T* cells, it is advisable to sort *T* cells from the spleen.

This study faced several limitations. While the effects of PTXF on TILs were investigated, the mechanisms by which PTXF affects TILs and exerts its antitumor effects were not fully evaluated. As mentioned earlier, PTXF improves oxygen delivery and dilates peripheral arteries [12]. Therefore, one potential mechanism by which PTXF could limit tumor growth and improve immune cell infiltration is by decreasing hypoxia or increasing the accessibility of immune cells to the tumor. Higher infiltration of immune cells in the TME following PTXF treatment might be a reason for better anti-tumor responses. The effects of PTXF on tumor hypoxia require further investigation. Our previous study on the ex vivo effects of PTXF on TILs showed that PTXF could directly decrease the regulatory TILs while increasing the antitumor CTL frequency and responses. Together with the present study, it could be concluded that the antitumor effects of PTXF on TILs might involve several in vivo and ex vivo mechanisms. PTXF may employ several mechanisms to limit tumor growth, including radiosensitization, chemosensitization, enhanced immune cell infiltration, inducing cell cycle arrest and apoptosis in cancer cells, and having anti-metastatic effects [13, 19–24]. Furthermore, emerging research suggests the potential synergistic benefits of combining pentoxifylline and histone deacetylase inhibitors (HDACi) in cancer treatment. This approach has demonstrated a significant reduction in tumor growth, even at lower doses of either drug when administered individually [33, 34]. These mechanisms should be investigated in the context of TNBC. Using transgenic mice, the main genes and proteins that play a central role in the antitumor effects of PTXF could be identified. Tregs may be one of the key players in this era, as our study showed their depletion following PTXF treatment.

Our findings suggest that the effect of PTXF on TILs was more significant than that of splenocytes. We believe that this finding might be an advantage of PTXF; because systemic depletion of Tregs may lead to concerns about inflammatory and autoimmune responses.

## Conclusion

Our study showed that pentoxifylline reduces the proportion of Tregs in the population of breast tumor-infiltrating lymphocytes and modifies the balance of cellular and cytokine responses in favor of antitumor responses. Pentoxifylline is a safe and well-tolerated drug widely used as an FDA-approved drug in cardiomyopathy, nephropathy, and vascular diseases [35, 36]. Due to its low toxicity profile [37, 38], the clinical use of this drug in antitumor therapies is much easier and more accessible than therapies that still have serious challenges and concerns about their safety [39, 40].

## Data Availability

Data of the study is available from the corresponding author on a reasonable request.
